# The Legal Implications of Report Back in Household Exposure Studies

**DOI:** 10.1289/EHP187

**Published:** 2016-05-06

**Authors:** Shaun A. Goho

**Affiliations:** Emmett Environmental Law and Policy Clinic, Harvard Law School, Harvard University, Cambridge, Massachusetts, USA

## Abstract

**Background:**

Scientists conducting research into household air or dust pollution must decide whether, when, and how to disclose to study participants their individual results. A variety of considerations factor into this decision, but one factor that has not received attention until now is the possibility that study participants’ receipt of their results might create legal duties under environmental, property, landlord–tenant, or other laws.

**Objectives:**

This article examines relevant laws and regulations and explores the scope of participants’ legal duties and the resulting legal and ethical consequences for researchers. Participants could be required in some situations to disclose the presence of certain chemicals when selling or renting their homes or to frequent visitors. The article discusses hypothetical case studies involving the reporting back of results regarding lead, polychlorinated biphenyls, and phthalates.

**Discussion:**

The potential legal duties of study participants have both ethical and legal implications for researchers. Issues include whether the legal consequences for participants should affect the decision whether to report back individual results, how researchers should disclose the legal risks to participants during the informed consent process, and whether researchers would be liable to study participants for legal or economic harm arising from reporting study results to them. The review provides recommendations for language that researchers could use in the informed consent process to disclose the legal risks.

**Conclusions:**

Researchers should still report back to participants who want to see their results, but they should disclose the risks of obtaining the information as part of the informed consent process.

**Citation:**

Goho SA. 2016. The legal implications of report back in household exposure studies. Environ Health Perspect 124:1662–1670; http://dx.doi.org/10.1289/EHP187

## Introduction

Because most people in the United States spend the vast majority of their time indoors, indoor environments are a major source of pollution exposure (Julien et al. 2008). Therefore, while public health research has traditionally focused on the impacts of outdoor pollution, in recent years increasing attention has been paid to exposure in indoor environments, such as homes, schools, and workplaces (Spengler and Adamkiewicz 2009).

This review refers to studies examining chemical exposures in homes, or “household exposure research.” These studies have demonstrated that household air and dust contain dozens of potentially harmful chemicals (Brody et al. 2009; Rudel et al. 2003; Mercier et al. 2011; Ashmore and Dimitroupoulou 2009; Weschler and Nazaroff 2008), including some that are heavily regulated or banned, such as lead, asbestos, and polychlorinated biphenyls (PCBs) (Arcury et al. 2014; Lu et al. 2013). Others, such as flame retardants, phthalates, or parabens, are approved for current use or are treated differently in different jurisdictions (Julien et al. 2008; Dodson et al. 2012b; Johnson et al. 2010; Allen et al. 2007; Bornehag et al. 2004; Su et al. 2013; Wilson et al. 2007). Yet as scientific evidence develops, some currently unregulated chemicals may in the future be regulated or even banned. These chemicals come from many sources, including combustion (gas stoves and ovens, furnaces, smoking), consumer products, building materials (including drywall, paint, varnishes, and caulking), the outdoor air, air from other units in a multifamily dwelling, and clothing brought home from a contaminated workplace (Rudel et al. 2003; Spengler and Adamkiewicz 2009).

Scientists engaged in household exposure research face several ethical decisions when deciding how to design their studies, including protocols for interacting with participants during recruiting, informed consent, and results reporting. Most fundamentally, researchers must ensure that the participants, as human subjects, are informed of the risks of participating in the study and voluntarily consent to take part in it. Other considerations include whether the researchers will engage in any follow-up testing to identify the sources of unusual contamination that is identified and/or attempt to remediate the contamination, and whether they can or will keep the participant’s results confidential.

Another major consideration is whether to provide participants with their individual results, a process known as “report back.” Some researchers and ethicists take the position that only clinically significant results should be reported because the report back of results with uncertain health implications will produce unnecessary fear and stress in study participants without any counterbalancing medical benefits. Others argue that researchers should generally share individual study results with participants in accordance with the ethical principle of respect for personal autonomy and to enable informed activism about community- or society-wide dangers such as local air pollution or harmful chemicals in consumer products. The latter position has gained increasing acceptance in recent years (Brody et al. 2014).

This article reviews laws and regulations that may have a major impact on the report-back decision—whether receiving individual results might trigger legal duties for study participants. For example, study participants who learn that their homes contain dangerous chemicals might have a legal duty to clean up the contamination or to report the presence of the chemicals to a government agency, home buyer, landlord, tenant, or visitor. Although the potential for legal consequences has been identified as a potential risk for study participants (Resnik 2012; NRC/IOM 2005), it has not previously been analyzed in depth.

Given the significant dangers associated with indoor air pollution and other indoor health hazards, it is important that there be no inappropriate or unplanned legal barriers to household exposure research (NRC/IOM 2005). The danger examined in this article is that environmental, public health, or other laws could have the perverse effect of hindering important research into indoor health hazards.

In this article, the author identified and reviewed legal duties under federal hazardous waste laws, and state hazardous waste, real estate transfer, landlord–tenant, and tort laws, that could be triggered by receiving study results.

## Results

The laws discussed in this article fall into three categories related to potential duties of study participants:

In a few limited circumstances, some laws create clear legal duties for study participants. These duties are generally limited to situations in which a participant learns about the presence of particularly dangerous and heavily regulated substances such as lead, asbestos, or PCBs.
A number of the laws create no duties for study participants because they contain exemptions for which the study participants would qualify.
Several laws result in unclear implications for study participants, either because they use open-ended language in which application of the law might change over time or because of other ambiguities.

As described in Table 1, three categories of laws—those with clear legal duties (defined as “Yes”), those that clearly impose no legal duties (defined as “No”) and those for which the answer is unclear (defined as “Uncertain”)—create unambiguous legal duties for participants in studies that were carried out in certain states and that involved the testing for particular substances:

**Table 1:** Laws applicable to study participants in household exposure studies.

Law(s)	Legal duties?	Chemicals	Details
Lead hazard act	Yes	Lead	Requires sellers or landlords of housing constructed before 1978 to include a U.S. Environmental Protection Agency (U.S. EPA)-approved Lead Warning Statement in sales or lease contracts and to disclose to buyers or tenants any known lead-based paint in the housing. Violators are subject to fines of up to $11,000 per violation (Lead Hazard Act 1992; Vidiksis *v*. Environmental Protection Agency 2010).
State laws pertaining to lead paint	Yes	Lead	Can impose requirements beyond federal standards. For example, in California, the State Department of Health Services or a local enforcement agency can order a property owner to abate a “lead hazard” caused by “lead-contaminated dust” [California Health and Safety Code 2015 §§ 17920.10(a), 105256(a)]. In Massachusetts, property owners must remove or cover loose lead paint and lead paint on windows and other surfaces accessible to children in any homes in which children under age 6 live (Massachusetts Lead Law, 2011, M.G.L. c. 111, § 197).
TSCA	Yes, but enforcement unlikely	PCBs	Makes it illegal for anyone to “manufacture, process, or distribute in commerce or use any polychlorinated biphenyl in any manner other than in a totally enclosed manner” [TSCA 1976, 15 U.S.C. § 2605(e)(2)(A)]. The U.S. EPA considers the continued use of materials, such as caulk, containing PCBs at concentrations > 50 ppm (parts per million) to be a violation of TSCA (U.S. EPA 2015).
State real estate transfer laws—specific provisions	Yes	Lead, asbestos, PCBs, pesticides	Thirty-six states mandate the use of forms that require home sellers to make certain disclosures to potential buyers. Some of these forms list specific substances or categories of substances that must be disclosed when present on the property. Lead and asbestos: almost all states (except in Virginia, where they are omitted from the mandated form, and in Idaho and Nebraska, where the statutory form does not include them but the form typically used by realtors does). Pesticides: Arizona, Colorado, New Jersey, New York. PCBs: Indiana, New Jersey, Pennsylvania. Must disclose that the property has been tested for hazardous substances: Delaware, Georgia, Nebraska, New Jersey, New York, Pennsylvania, South Dakota.
CERCLA	No	Various	Participants would be exempt for the following reasons: *a*) the statute defines “environment” as “water…, land surface or subsurface strata, or ambient air within the United States or under the jurisdiction of the United States” [CERCLA 1980, 42 U.S.C. § 9601(8)], and most courts have taken the position that “the ‘environment’ referred to in the statute ‘includes the atmosphere, external to the building,’ but not the air within a building” (3550 Stevens Creek Associates *v*. Barclays Bank of California 1990; Fertilizer Institute *v*. U.S. EPA 1991); and *b*) although many of the chemicals tested for in household exposure studies are controlled as hazardous substances, the threshold quantities identified in the U.S. EPA’s regulations are much higher than would likely be found in a home [e.g., the reportable quantity of diethyl phthalate is 1,000 pounds (U.S. EPA 2015a)].
EPCRA	No	Various	Participants would be exempt because the threshold levels of “extremely hazardous substances” needed to trigger responsibilities under this law are greater than any individual would have in his or her home [EPCRA 1986, 42 U.S.C. § 11002(a)].
RCRA	No	Various	Participants would be exempt because chemicals that come from products in use have not been discarded and therefore are not waste for purposes of RCRA (Safe Air for Everyone *v*. Meyer 2004) and because the U.S. EPA has exempted household waste from the definition of hazardous waste (U.S. EPA 2015b).
State laws pertaining to hazardous waste	No	Various	Contain exemptions similar to those under CERCLA, EPCRA, and RCRA. For example, under the Massachusetts Hazardous Waste Management Act, anything not discarded is not “waste” (CMR 2015b § 30.010) and household waste is excluded from the definition of hazardous waste [CMR 2015b § 30.104(2)(g)]. Under the Massachusetts Oil and Hazardous Material Release Prevention Act (2008), only a “release…into the environment” need be reported. Similar definitions and exemptions have been adopted by most states.
State real estate transfer laws—catch-all provisions	Uncertain	Various	Many states require sellers to disclose the presence of “environmental hazards.” Substances that would need to be disclosed are probably limited to those *a*) with significant impacts on human health, *b*) that are heavily regulated or banned, and *c*) for which a primary means of exposure is indoor air as a result of the chemicals’ presence in the building or its fixtures.
Duty to disclose latent defects	Uncertain	Various	Sellers and landlords must disclose hidden defects. Flaking lead paint, for example, has been identified as a latent defect that must be disclosed (Flowers *v*. ERA Unique Real Estate, Inc. 2002). Some older decisions also held landlords liable for failing to disclose the existence of certain contagious diseases in their rental properties (Leech *v*. Husbands 1930; Minor *v*. Sharon 1873). By analogy, a landlord or seller might be held liable for failing to disclose a hazardous chemical present at a concentration high enough to cause illness or injury.
Implied warranty of habitability	Uncertain	Various	In all states except Arkansas, the law imposes on all residential leases a guarantee (or warranty) from the landlord to the tenant that the property is in habitable condition. To violate this implied warranty of habitability (IWH), an apartment does not need to be literally uninhabitable; “[g]enerally, a defect is considered actionable if it renders the premises unsafe or unsanitary” (Lonegrass 2010). In California and Massachusetts, for example, a landlord’s knowledge that a property contains lead paint can be a violation of the IWH (California Civil Code 2015 § 1941.1; California Health and Safety Code 2015 § 17920.10; Chase *v*. Pistolese 2002; Elliott *v*. Chaouche 2000; CMR 2015a § 410.750). A Massachusetts court, in reaching this decision, relied on a regulation that identified the presence of lead paint as a violation of the state sanitary code. Because the presence of asbestos dust is also a violation of the sanitary code, the presence of asbestos could also violate the IWH. By analogy with the real estate disclosure forms discussed above, it is probable that only chemicals of similarly proven harmfulness would require remediation under this standard.
Tenants’ disclosure duties	Uncertain	Various	Tenants must keep premises in a “safe and sanitary” condition. California requires that tenants notify landlord of “any release of a hazardous substance.”
Premises liability	Uncertain	Various	In some situations, owners must warn visitors of “hazards,” “defects,” or “dangerous” or “unsafe” conditions on their property.
Note: A single § refers to one section in a statute; §§ refers to multiple sections.

Both federal and state laws have created duties for homeowners who learn that their homes contain lead paint. Federal law requires that they disclose the presence of the lead paint to any potential buyers or renters. State law can, in some circumstances, require homeowners to remove or cover the lead paint. The report back of results identifying the presence of lead in dust is discussed in the “Case Studies” section in this article.
The use of PCBs was banned under the Toxic Substances Control Act of 1976 (TSCA 1976). The U.S. Environmental Protection Agency (EPA) considers the continued use of materials containing PCBs, such as caulk, at concentrations > 50 ppm (parts per million) to be a violation of TSCA (U.S. EPA 2015c).
Study participants would need to disclose certain results on some of the property disclosure forms that many states require homeowners to fill out and provide to prospective buyers. The disclosure forms typically ask sellers to reveal any knowledge they have of flaws in the home, including structural problems, water damage, and pest infestations. Several chemicals and groups of chemicals that have been the subject of household exposure studies—lead, asbestos, pesticides, and PCBs—are specifically listed on some real estate disclosure forms. In addition, seven states require sellers to disclose on the real estate transfer disclosure forms whether their property has been tested for hazardous substances.

There are also laws that do not impose duties on study participants. In particular, federal and state hazardous waste laws, which address the generation, storage, disposal, and releases of chemicals that are sometimes included in household exposure studies, contain exemptions applicable to study participants. Under the Comprehensive Environmental Response, Compensation, and Liability Act of 1980 (commonly known as Superfund) (CERCLA 1980), any person in charge of a “facility” must report to the National Response Center if a certain quantity of a “hazardous substance” is “released” from the facility “into the environment” (CERCLA 1980, 42 U.S.C. §§ 9602–9603). The presence of a chemical in indoor air or dust, however, does not indicate that a release into the “environment” has occurred and the threshold quantities identified in EPA’s regulations are much higher than would likely be found in a home. Under the Emergency Planning and Community Right to Know Act of 1986 (EPCRA 1986), facilities containing certain chemicals in quantities above threshold levels must report the chemicals’ presence to the state emergency response commission (U.S. EPA 2015d). As with CERCLA, no study participant would have chemicals present in their homes in sufficient quantities to trigger a duty to report. The Resource Conservation and Recovery Act of 1976 (RCRA 1976) regulates the generation, transportation, storage, and disposal of hazardous wastes. Participants are exempt from its requirements because chemicals that come from products in use have not been “discarded” and therefore are not “waste” for purposes of RCRA (Safe Air for Everyone *v*. Meyer 2004) and because the U.S. EPA has exempted household waste from the definition of hazardous waste (U.S. EPA 2015b). State hazardous waste laws are largely modeled on their federal counterparts and contain similar exemptions. The effect of these exemptions is that most chemicals present in homes, or disposed of through regular trash collection, are exempt from reporting or other requirements under state hazardous waste laws.

Five types of laws, however, have unclear consequences:

Real estate transfer laws—catch-all provisions—Many states have real estate transfer forms that have some kind of catch-all provision that typically asks if the seller is aware of any substances, materials, or products, which may be an environmental hazard, and then lists some examples of hazardous chemicals or substances (Table 1). The identified chemicals may include asbestos, lead paint, urea formaldehyde, radon gas, fuel or chemical storage tanks, or contaminated soil. To predict how a court or agency would interpret the catch-all provisions when faced with the findings of a household exposure study, it is therefore helpful to analyze the text of a typical provision. California’s form is representative of those of many other states in that it requires the disclosure of “[s]ubstances, materials, or products which may be an environmental hazard such as, but not limited to, asbestos, formaldehyde, radon gas, lead-based paint, mold, fuel or chemical storage tanks” (California Civil Code 2015 §1102.6). Under a principle of statutory interpretation, known in lawyers’ Latin as *noscitur a sociis*, when a legal document contains a general class of items, followed by a list of examples of the class, the character of the general class is informed by the nature of the listed items. “[T]he most common effect of the canon is…to limit a general term to a subset of all the things or actions it covers” (Scalia and Garner 2012). The general class here is “environmental hazards,” and this class is limited by the listed substances, such as asbestos, formaldehyde, radon gas, and lead-based paint.

These substances share some characteristics. First, they are well known to have significant impacts on human health, including developmental delays, lung disease, and increased risk of some cancers (Markowitz and Rosner 2000; Bartrip 2004; NCI 2011a, 2011b). Second, they are generally heavily regulated or banned [e.g., lead paint (CPSC 1977), asbestos (U.S. EPA 1999), and urea formaldehyde foam insulation (CPSC 1982)]. Radon is the exception, because its source is naturally occurring decay of radioactive elements in rocks and soil, rather than any consumer products. Third, one of the leading sources of exposure to all of these chemicals is indoor air. In particular, the primary source of the chemicals in a home is the building itself or its fixtures (or in the case of radon the ground under the house) rather than in consumer products or furniture that would be removed when the current residents leave the house. A plausible interpretation of these provisions, therefore, would limit them to chemicals that share these three characteristics. The application of these provisions to different chemicals is discussed in the “Case Studies” section.

Duty to disclose latent defects—A second type of law is the duty of both sellers and landlords to warn prospective buyers and renters, respectively, of hidden, or latent, defects (Lord 2015; Moynihan and Kurtz 2005). Under this doctrine, a participant would likely need to disclose only chemicals present at a concentration high enough to cause illness or injury.
Implied warranty of habitability (IWH)—Landlords are subject to an IWH in residential leases, under which the landlord warrants that there are no defects vital to the residential use of a unit and that the premises will remain livable throughout the tenant’s occupancy. It is likely that only the presence of lead paint, asbestos, or other chemicals of similarly proven harmfulness would require remediation under this doctrine.
Tenants’ disclosure duties—Tenants also have potentially relevant legal duties. For example, they must keep their premises in a safe and sanitary condition (Ohio Revised Code 2015; Zito *v*. 241 Church St. Corporation 1996). It is unlikely that a study participant who is a tenant would owe any duties to a landlord under this requirement. On the one hand, chemicals originating from the fixtures of the home would be the landlord’s responsibility, not the tenant’s. Any chemicals detected in a study will either originate from the fixtures of the home (and therefore be the landlord’s responsibility) or from the tenant’s personal property (and therefore any traces left in the air or dust will dissipate after the tenant moves out). Either way, there will be no lingering harm that is the tenant's responsibility.

In addition to the general rule to keep the premises safe and sanitary, California requires that tenants notify their landlord if there is “any release of a hazardous substance” on the property [California Health and Safety Code 2015 § 25359.7(b)]. If the tenant fails to make the required notice, the landlord can void the lease. This requirement is potentially problematic for study participants, because it contains no *de minimus* exception. As noted by the authors of one treatise: “Literal construction of this statute would allow the landlord to terminate the lease because tenant failed to provide written notice that he spilled common hazardous substances such as solvents, paint, photocopier fluid, or bleach on the premises” (Machlin and Young 2014). Given the extreme consequences of applying the statute in this way, however, it is possible that a court would read into it an exception. It can also be argued that the receipt of study results, which merely indicates the presence of chemicals, and not their source or the timing of any release, does not trigger this notification requirement.

Premises liability—A final area of law to consider is the duties that property owners or occupiers owe to visitors, a topic known as premises liability. In some situations, owners must warn visitors of hazards, defects, or dangerous, or unsafe conditions on their property. It is conceivable that a study participant’s knowledge of the presence of certain chemicals could trigger a duty to warn visitors.

Successful premises liability claims have been won by employees who were exposed to indoor air pollution in commercial buildings. For example, in one case, U.S. EPA employees who developed neurological illnesses after the agency’s Washington, DC, headquarters was renovated in the late 1980s were able to win damages in a suit against the building’s owner (Bahura *v*. S.E.W. Investors 2000). In another case, an employee in an office building sued the landlord after suffering “headaches, dizziness, nausea and blurred vision, as well as damage to her brain and central nervous system” as a result of exposure to volatile organic compounds (VOCs) that originated in “the materials used in the construction and decoration of the building” (Mackey *v*. TKCC, Inc. 1995).

Study participants, however, are unlikely to be liable to a visitor except in extreme circumstances. The possibility that a short-term visitor, even a repeated one, would suffer an injury that was caused by chemicals present on the property would usually be remote. Such an outcome might be possible for some of the most dangerous chemicals that could be found on a property, such as lead or asbestos, but even then only if the visitor’s activities actually brought her into contact with the substance. In addition, such an outcome would require the study participant, who actually lives in the home, to do nothing when notified about a condition so dangerous that it could harm even a short-term visitor to the property. On the whole, such a set of circumstances seems unlikely, though not completely impossible.

## Case Studies

To provide more specific guidance for researchers, this section applies the legal rules to three case studies.

### Lead

Most laws regulating lead in households refer to lead paint, rather than lead in the air or dust. Therefore, the presence of lead in the air or dust does not necessarily trigger any legal duties. One exception is in California where the California Health and Safety Code identified “lead-contaminated dust” as a violation [California Health and Safety Code 2015 § 17920.10(a)]. Even this law establishes thresholds (e.g., 40 μg/ft^2^) for interior floor surfaces. Depending on the sampling methodology used in the study, it might not be possible to determine whether this threshold has been exceeded.

If lead is found at a high concentration in air or dust from a home built before 1978, however, it can be inferred that lead paint is the likely source of the lead contamination. In response to such a finding, a whole host of legal consequences would follow. If the participant wanted to sell or lease the home, under the federal Lead Hazard Act she would need to disclose the presence of lead paint to the buyer or renter [Residential Lead-Based Paint Hazard Reduction Act of 1992 (2009), 42 U.S.C. § 4852d]. Most states also require the disclosure of lead paint in their real estate transfer disclosure forms. If the participant is a landlord, the IWH and duty to disclose latent defects would provide an independent basis for requiring the disclosure of the presence of lead paint to existing or potential tenants. All participants (not only those who are landlords) would also need to disclose the presence of lead paint to visitors to avoid premises liability, particularly if it is reasonable to believe that the visitors (based on the frequency of visits or their susceptibility, as with young children) might be harmed. Depending on the state in which the participant lives and/or whether young children live in the home, state laws might require that she remove or cover the paint.

### PCBs

Four of the categories of laws—TSCA, real estate transfer disclosure forms, landlord–tenant laws, and premises liability—are potentially implicated by a finding of PCBs in household air or dust.

The U.S. EPA has interpreted TSCA to mean that the presence of PCBs at a concentration greater than 50 ppm in building materials such as caulk is a violation of TSCA. However, the detection of PCBs in an air or dust sample does not indicate the source of the PCBs or the concentration of the PCBs in that source. Therefore, without follow-up testing, it is not clear that participants who receive their results would know of a TSCA violation.

Even if the researchers carried out follow-up testing and identified a source material that contained PCBs at a concentration > 50 ppm, it is unlikely that the U.S. EPA would bring an enforcement action against the study participant. The U.S. EPA has stated that it “believes that enforcement may not be the most effective tool to reduce health risks” when PCBs are identified in schools and other buildings and that “such buildings will in most cases be a low priority for enforcement” (U.S. EPA 2015c).

Three states—Indiana, New Jersey, and Pennsylvania—specifically require the disclosure of PCBs on real estate transfer disclosure forms, and in these states study participants would need to disclose the presence of PCBs when selling their homes.

In addition, many states include a catch-all provision on their disclosure forms that refers to environmental hazards or a similar term. PCBs share many characteristics with the chemicals listed on these forms, such as asbestos, formaldehyde, and lead-based paint. PCBs have been banned in the United States since 1979 (U.S. EPA 1979), and the U.S. EPA (2016) classifies PCBs as “probable human carcinogens.” As with the listed chemicals, indoor air is thought to be a significant exposure pathway, although the most significant is the consumption of contaminated food (Rudel and Perovich 2009). In addition, the most likely sources for PCBs found in air or dust are building materials such as paint, caulk, plaster, or floor finishes.

The real estate transfer forms do not specify any minimum concentration of PCBs necessary to trigger the disclosure requirement. Read literally, therefore, these forms require study participants to disclose to potential buyers the presence of PCBs at any concentration, however low. Such an outcome would be problematic, as a significant percentage of samples in a study can detect PCBs. For example, one study found PCBs in 31% of the 120 homes tested in Cape Cod, Massachusetts (Rudel et al. 2008). Another study detected PCBs in 6 out of 10 apartments tested in Davis, California (Hwang et al. 2008). It seems more reasonable to conclude that the presence of PCBs represents an environmental hazard or hazardous conditions only when they are detected at high levels.

Similarly, an unusually high concentration of PCBs in household air or dust would likely need to be disclosed by a landlord to potential tenants as a latent defect or need to be cleaned up as a potential violation of the IWH. Finally, repeated visitors, particularly those who might come into contact with contaminated dust, such as workers who use floor sanders on a hardwood floor with a contaminated finish, would likely need to be warned.

For a real-world example of the detection of PCBs, Rudel et al. (2008) identified two homes with much higher levels of PCBs than in the other homes in their Cape Cod study. Subsequent testing and interviews with residents led researchers to an inference that the source of the PCBs was a floor finish used in the 1950s. However, the researchers did not know whether any surface (such as floors and walls) had PCBs at a concentration > 50 ppm. Massachusetts does not have a mandatory real estate transfer disclosure form. Given the unusually high concentrations of PCBs in these homes, and the high levels of PCBs that the residents had in their blood, however, the participants likely would have needed to disclose the PCBs as a latent defect to a potential buyer or renter.

### Polybrominated Diphenyl Ethers (PBDEs) and Phthalates

Unlike lead or PCBs, no federal or state laws have created specific disclosure or remediation duties for PBDEs. Moreover, no real estate transfer disclosure forms specifically identify them. The questions then are whether they would be covered by general environmental hazard provisions on the disclosure forms and whether their presence would need to be disclosed under landlord and tenant laws or premises liability laws.

The chemicals that are listed as examples of environmental hazards on disclosure forms are typically substances that are prohibited from use, are known carcinogens, and for which building materials used in residential homes (such as caulk and paint) are major pathways of exposure. Under these criteria, the presence of PBDEs and phthalates would not currently need to be disclosed on such forms. First, both types of chemicals are present in many consumer products currently in use. Although the use of PBDEs as flame retardants in new consumer products in the United States has been largely phased out over the last decade, they continue to be present in many in-use mattresses, upholstered furniture, electronics, and fabrics (Abbasi et al. 2015). Phthalates are currently used in plastics, cosmetics, perfumes, and pesticides—one study found that vinyl shower curtains were 28% Bis(2-ethylhexyl) phthalate (DEHP) (Dodson et al. 2012a). Second, the evidence of harm from PBDEs and phthalates is not yet as strong as it is for lead, asbestos, or PCBs. Third, the primary source of PBDEs and phthalates could be consumer products, which study participants would take with them if they moved out of their homes. It is thus not clear that the presence of these chemicals in household air or dust, as revealed by a household exposure study, indicates the presence of the chemicals in the “property” that is to be transferred.

The situation is not as clear-cut as this brief summary makes it appear, however. First, although products containing PBDEs and phthalates are still widely used, 12 states and the District of Columbia have banned the use of pentaBDE, octaBDE, and/or decaBDE in some consumer products (Corrigan 2016), while the federal government has banned the sale of children’s products containing more that 0.1% DEHP, dibutyl phthalate (DBP), or benzyl butyl phthalate (BBP) (Consumer Product Safety Act 2015).

Second, although the evidence of harm may not be as well established as for lead or asbestos, there is rapidly accumulating evidence of harmful effects from PBDEs and phthalates. PBDEs “have been associated with liver toxicity, thyroid toxicity, and neurodevelopmental toxicity in humans” (Corrigan 2016). Phthalates are associated with endocrine disruption, male infertility, and respiratory symptoms. A recent study estimated the economic costs of male reproductive disorders and diseases attributable to PBDEs and phthalates in the European Union as approximately €15 billion per year (Hauser et al. 2015).

Third, although consumer products might be the main source of exposure, both PBDEs and phthalates can be used in building materials and other fixtures that will remain in the home after a study participant moves out. PBDEs are in insulation, carpeting, lamp sockets, kitchen hoods, and pipes. Phthalates, in turn, are used in vinyl flooring and various building materials that include polyvinyl chloride (PVC).

Therefore, a study participant would likely not need to disclose the presence of chemicals like phthalates and PBDEs, either when selling a home or to tenants or visitors. However, both the scientific evidence and regulatory status of these chemicals are in flux, so the strength of the argument for requiring disclosure could increase in the future.

Chlordane, an insecticide that was widely used for termite control from the 1950s to the 1980s, is an example of the way in which the status of a chemical can change relatively rapidly. It was approved for use on food crops until 1978, continued to be used as a termite treatment through most of the 1980s, and was only banned completely in 1988. By the early 1990s, however, a number of lawsuits had been filed by residents of houses and apartment buildings that had been treated with chlordane (Thornton *v*. Fondren Green Apartments 1992; Kornreich 1990). In at least one case, a buyer tried, unsuccessfully, to get a court to undo a home sale on the grounds that the seller had failed to inform the buyer that the home had been treated with chlordane (Copland *v*. Nathaniel 1995).

## Discussion

As described above, study participants may need to disclose the results of household exposure studies to third parties in some circumstances. The strongest cases for disclosure are when the chemicals identified are banned substances such as lead paint, asbestos, or PCBs. High levels of other chemicals, such as phthalates and PBDEs, likely do not need to be disclosed now, but might in the future, as scientific understanding of the harmfulness of the studied chemicals advances and as their regulatory status changes.

### The Decision Whether to Report Back

These findings raise several legal and ethical issues for researchers. The first and most basic is whether they should affect researchers’ decision whether to report back individual results to participants. The standard ethical framework for such decisions is derived from the Belmont Report, produced in 1979 by the National Commission for the Protection of Human Subjects of Biomedical and Behavioral Research. It identified three basic ethical principles to govern human subjects research: *a*) respect for persons, *b*) beneficence, and *c*) justice (Department of Health, Education, and Welfare 1979). Respect for persons means that researchers should respect the autonomy of individuals and their independent decisions. Beneficence requires that researchers do no harm and maximize benefits for human subjects. Finally, justice requires that researchers ensure a fair distribution of the benefits and burdens of the research.

The Belmont principles are embodied in the Federal Policy for the Protection of Human Research Subjects (DHHS 2015)—known as the “Common Rule” because it has been adopted by 17 federal agencies. Institutions can also choose to provide “assurances” to the federal government that they will comply with the Common Rule. Any research carried out on household exposures, either with federal funding or at an institution that has made an assurance of compliance, will therefore be subject to the Common Rule. Other research, however, is not covered.

There has been controversy about when, according to these principles, researchers should report back individual results to study participants. Under the traditional, clinical model of biomedical research, researchers do not provide individual results to participants unless those results are clinically significant. Underlying this view is the concern that a participant who receives study results whose medical significance is unclear will be subject to needless worry without any countervailing benefit and therefore that report-back is inconsistent with the principle of beneficence (Deck and Kosatsky 1999; Miller et al. 2008). Given the substantial uncertainties surrounding the extent, nature, and conditions that cause harm from environmental chemical exposures, much of the data generated by household exposure studies would not qualify for report-back under this standard.

Others, however, argue that researchers should generally share individual study results with participants who want them. Advocates of this position argue that this approach better serves the “respect for persons” principle (Shalowitz and Miller 2005). They also observe that a growing body of empirical research indicates that participants want to receive their individual results and do not react with undue alarm (Brody et al. 2014; Altman et al. 2008). As a result, some writers have suggested that researchers working with human subjects have an ethical and/or legal duty to provide subjects with the choice of whether to learn their individual results (Gordon 2009; Shalowitz and Miller 2005). In the context of biomonitoring, this view has been endorsed by several prominent organizations and committees (Brody et al. 2014).

It has been suggested that the sorts of legal risks discussed here might provide a reason not to report back the results of household exposure studies (Resnik 2012). The results of this review, however, suggest that the legal risks are not so grave that researchers should decline to report such results. First, in most cases, report-back will not trigger any legal duties for participants; in those situations, it presents no risk. Second, the rare cases when participants will be legally required to disclose and/or remediate chemical contamination identified in the study will generally also be situations in which the identified chemicals (such as lead, PCBs, or chlordane) could be harmful to the study participant as well as other residents of the home. The benefits of receiving the results in such situations likely outweigh the risks: If participants receive these results, they might be able to take actions to reduce their own exposure to the chemicals. In fact, researchers may actually have a duty to warn the participants when study results indicate the existence of a significant health risk (Resnik and Zeldin 2008; Grimes *v.* Kennedy Krieger Institute, Inc. 2001).

When there is some uncertainty about the magnitude and nature of the legal risk, researchers can minimize the potential harm to participants through a well-thought-out report-back process. The report-back package should include contextual information. For example, it could allow participants to compare their results to those of other homes (either from the same study or from other studies) and to relevant regulatory benchmarks. If a participant’s results do not indicate abnormally high levels of especially dangerous chemicals, then even if she is subsequently obliged to turn over the results to a potential buyer, renter, or other person, the contextual information should prevent such disclosure from causing undue alarm. For example, if a participant in Indiana, New Jersey, or Pennsylvania learns that a sample taken in her home contains PCBs, she would be obliged to disclose this fact when selling her home. If the level of PCBs detected was similar to that in other homes in the study and/or below relevant regulatory thresholds, then any harm from having to disclose this fact would probably be minimal.

Researchers also should be prepared to carry out follow-up testing to identify the sources of unusually high concentrations of chemicals detected in initial tests. Consider the example in which unexpectedly high levels of PCBs were identified in two homes in Massachusetts (Rudel et al. 2008). Additional testing and investigation in that case revealed a previously unknown source of PCB exposure. This type of outcome can help participants minimize their risk of harm and add to scientific knowledge about the sources of environmental chemical exposures (Morello-Frosch et al. 2015).

Study participants always have the option of choosing not to receive their results. Advocates of reporting back never suggest that participants should be compelled to receive their individual results. Rather, they stress the principles of justice and respect for persons and that the participants, rather than the researchers, should make the final choice. If participants are concerned about the potential legal consequences, then they can choose not to receive their results.

### Informed Consent

Researchers can also minimize the risk of harm to participants through the informed consent process. One of the fundamental requirements of human subjects research is that the participation of human subjects must be voluntary. The concept of voluntary participation is embodied in the requirement of informed consent, which is meant to ensure that researchers provide participants with all of the information they need to make an independent decision about whether to participate in the study after weighing all of the costs and benefits (Korobkin 2007).

The Common Rule requires that, as part of the informed consent process, researchers must provide human subjects with a “description of any reasonably foreseeable risks or discomforts to the subject” [DHHS 2015 § 46.116(a)]. Most discussions of the risks that need to be disclosed focus on the physical harms that might arise from a medical intervention (Reilly et al. 1997). Legal or economic harms, like those that might arise from receiving household exposure study results, are different in character. Nevertheless, it is now widely acknowledged that the risk of discrimination in health insurance and employment should be disclosed to individuals who participate in genetic research (Reilly et al. 1997; National Bioethics Advisory Commission 1999). By analogy, the legal risks identified in this paper should also be disclosed in the informed consent process.

If researchers must include information about legal risks in their informed consent documents, they will face challenges. Disclosing such information might discourage potential participants from taking part in the study. It is difficult to know how significant a problem this might be. It could probably be minimized by providing contextual information and offering follow-up testing to identify the source of any anomalous results, as described above. They also will face difficulties in developing the appropriate language for the informed consent documents. Identifying and assessing the risks requires legal expertise that will not typically be held by any member of the research team. Moreover, the specific legal risks will vary from state to state and will also depend on the particular substances being tested for in the study. The risks may also change over time, as new statutes, regulations, or judicial decisions are issued.

The following two-pronged approach might help. For example, if researchers know that their analyte list includes lead, asbestos, chlordane, PCBs, and/or formaldehyde, then the informed consent materials should notify participants that the detection of these substances could create a legal duty to notify potential buyers and renters, as well as visitors who would have a particularly high risk of exposure. However, if the analyte list does not include these chemicals, then the documents could indicate that there will generally be no legal risk for the participant, unless the testing produces a highly unusual result. For example, the informed consent documents might include a statement that includes the following information:

WHAT ARE THE RISKS OF PARTICIPATING IN THIS STUDY? This study will identify whether certain chemicals are present in the air or dust of your home and, if so, at what concentrations. If you choose to receive your individual study results, there is a very small chance that these tests will reveal the presence of a harmful chemical at such a high concentration that you would be under a legal duty to disclose the test results to someone else—for example, to a potential buyer if you are a homeowner and later decide to sell your home.

In addition to the general statements in the informed consent documents, researchers could provide participants with additional information on legal risks in an appendix to the informed consent document. In this way, the text of the basic consent document could remain short and clear, but participants would have access to additional detail. This approach would be consistent with the recently issued proposed revisions to §_.116 of the Common Rule (DHS et al. 2015).

More generally, researchers and institutional review boards can consult with the university’s general counsel if they need additional information about the legal risks presented by a particular study. The general counsel’s office could help draft the more detailed description of the legal risks that would be included in the appendix to the informed consent document.

Although the details of the legal issues are complex, the simple two-pronged approach described above should convey the essential information to participants in a relatively easy-to-understand manner. In addition, these legal issues are likely no more difficult for participants to understand than the complex medical and scientific issues that are routinely described in informed consent documents. In any event, these issues do not affect a participant’s decision to take part in a study, but only affect the decision whether to receive individual results.

### Researchers’ Potential Liability

If participants suffer any legal harms as a result of receiving study results, they might sue the researchers to recover for their losses. In general, it seems unlikely that researchers will be liable to participants for harms, particularly legal harms, arising from the sharing of study results—especially if they disclose the risks in advance. This issue is unclear, however, both because laws regulating researcher conduct are not designed to address this specific question and because only one state—Maryland—has established that researchers have specific duties of care. In Grimes *v*. Kennedy Krieger Institute, Inc. (2001), the Maryland Court of Appeals held that researchers have certain responsibilities to participants in non-therapeutic research. In particular, the Court held that researchers have a duty to disclose material information in the informed consent process. The court also stated that in non-therapeutic studies, researchers have a duty to protect participants from unreasonable harm and to promptly inform participants of potential hazards of the study. Because no other court has recognized these duties, they are binding only in Maryland and do not directly affect researchers working elsewhere.

The duties outlined in Grimes *v*. Kennedy (2001) focused on protecting the participants from physical harm arising from the nature of the research (the study in question examined different lead paint remediation techniques), not from potential legal risks indirectly stemming from the report-back of results. The court emphasized that its holding was applicable “when researchers recruit people, especially children whose consent is furnished indirectly, to participate in non-therapeutic procedures that are potentially hazardous, dangerous, or deleterious to their health” (Grimes *v*. Kennedy Krieger Institute, Inc. 2001). A legal risk arising from disclosure of results to participants is a more attenuated form of harm than a health hazard that is caused by the research itself. Therefore, even in Maryland, it is possible that a court would not find that researchers have a duty to protect participants from indirect legal harms resulting from the disclosure of results.

Even if researchers do owe participants a duty of care regarding legal risks, it seems likely that in this context a researcher would satisfy this duty by disclosing the risks in the informed consent process. If a participant, knowing the potential legal risks, agrees to receive the study results, it is hard to see how a researcher could be found negligent merely for providing those results.

Conversely, if a study participant chose not to receive her individual results, but the researchers later discovered high levels of a clearly hazardous chemical in those results, then that situation presents a much closer analogy to the Grimes *v*. Kennedy case. Researchers likely have an ethical duty to report the results under these circumstances, despite the participant’s initial refusal (Resnik and Zeldin 2008), and in Maryland, would have a legal duty to do so as well. They would have no legal duty to report in other states, unless the courts of those states choose to follow the Grimes *v*. Kennedy decision.

## Conclusions

Household exposure research is an important and growing field. Because of its importance, such research should not be inhibited by unnecessary legal barriers. At the same time, however, the research must be done in a way that does not expose study participants to significant risks without the knowledge and consent of the participants.

This article has examined one potential risk to participants: Receipt of their individual study results will create a duty to disclose the results to third parties, including government regulators, homebuyers, or tenants. It concludes that these risks are real, though limited, and that researchers should therefore disclose these risks as part of the informed consent process. The risks of compelled disclosure do not mean that researchers who conduct household exposure studies should refrain from reporting back to their study participants. There are significant benefits from the report-back process and these outweigh the potential harm identified.
